# Machine learning-based association analysis of triglyceride-glucose index with melanoma prevalence and all-cause mortality: insights from cross-sectional NHANES 1999–2018 data and an external hospital-based dataset

**DOI:** 10.3389/fnut.2026.1726865

**Published:** 2026-03-18

**Authors:** Yao Liang, Kaize Lin, Xunyi Long, Yun Wang, Chengxu Liu, Chenghan Xie, Qianye Wu, Dandan Li, Baiwei Zhao

**Affiliations:** 1 State Key Laboratory of Oncology in South China, Guangdong Provincial Clinical Research Center for Cancer, Collaborative Innovation Center for Cancer Medicine, Sun Yat-sen University Cancer Center, Guangzhou, China; 2 Department of Gastric and Melanoma Surgery, Sun Yat-sen University Cancer Center, Guangzhou, China; 3 Zhongshan School of Medicine, Sun Yat-sen University, Guangzhou, China; 4 Biotherapy Center, Sun Yat-sen University Cancer Center, Guangzhou, China

**Keywords:** all-cause mortality, machine learning, melanoma, subgroup analyses, TyG index

## Abstract

**Background:**

Insulin resistance has been associated with melanoma, however, the relationship between the triglyceride-glucose (TyG) index and this condition remains unclear. This study aims to investigate the relationship between the TyG index and melanoma.

**Methods:**

This study included 21,360 participants from the 1999–2018 National Health and Nutrition Examination Survey (NHANES). We used weighted logistic regression for the TyG index's link with melanoma prevalence, weighted Cox regression for mortality, restricted cubic spline for dose-response, and subgroup analyses to verify robustness. An optimal predictive model was constructed using seven machine learning algorithms, and Shapley additive explanations (SHAP) values visualization was conducted. A total of 475 patients with primary non-metastatic acral melanoma from three tertiary hospitals were included for descriptive analysis.

**Results:**

After demographic adjustment, the third TyG tertile exhibited elevated all-cause mortality risk (HR: 1.329, 95% CI: 1.201–1.458, *P* < 0.01). However, after further adjustment for all covariates, this association was no longer statistically significant (*P* > 0.05). RCS demonstrated a U-shaped relationship between the TyG index and all-cause mortality. Similar results were observed across most subgroup analyses. The ridge regression model (AUROC = 0.85) performed optimally, with SHAP analysis identifying race, age, and serum phosphorus levels as key predictors of melanoma risk.

**Conclusion:**

The TyG index shows a U-shaped association with all-cause mortality in patients with melanoma. The ridge regression model demonstrated the best predictive performance for melanoma risk in internal validation, with SHAP analysis identifying race, age, and metabolic markers as key influencing factors.

## 1 Introduction

Melanoma, which originates from melanocytes, is a malignant tumor that occurs primarily in the skin. It is characterized by high aggressiveness, predisposition to metastasis, and high lethality, accounting for more than 75% of skin cancer-related mortality (1, 2). Beyond its high mortality, melanoma often leads to severe clinical consequences, significantly impacting patients' quality of life and imposing substantial healthcare burdens and costs. Despite advances in immunotherapy and targeted therapy that have improved survival outcomes for some patients, the high metastatic potential and heterogeneity of melanoma still result in considerable variability in prognosis (3). These challenges underscore the urgent need to explore additional risk factors influencing melanoma progression, which may provide new scientific insights to reduce melanoma-related mortality.

Historically, research on melanoma has focused primarily on risk factors such as ultraviolet radiation exposure (4), as well as the roles of antioxidants (5) and inflammatory markers (6). However, recent studies have begun to uncover a link between melanoma and metabolic dysregulation (7). For instance, investigations into diabetes and altered glycolysis pathways suggest broader metabolic involvement in melanoma development and progression (8), Notably, insulin resistance (IR) has emerged as a potential contributor to melanoma metastasis, with metabolic syndrome playing a key role in this association (9). The triglyceride-glucose (TyG) index, a novel and reliable biomarker for assessing IR and metabolic syndrome (10, 11), has been associated with various chronic conditions (12), including cardiovascular disease (13–15), diabetes mellitus (16), and other dermatological conditions (17). However, the relationship between the TyG index and melanoma remains insufficiently explored.

To address this gap, we conducted a large-scale population-based study using data from the National Health and Nutrition Examination Survey (NHANES) spanning 1999–2018. NHANES provides nationally representative data, allowing for robust assessment of the TyG index's association with melanoma incidence and mortality.

Additionally, leveraging machine learning techniques, we developed a predictive model that integrates the TyG index with other clinically significant variables to enhance the accuracy of malignant melanoma risk assessment. This methodology enables the effective utilization of complex datasets to construct robust predictive models for melanoma risk.

## 2 Methods

### 2.1 Study design

**Figure 1:**
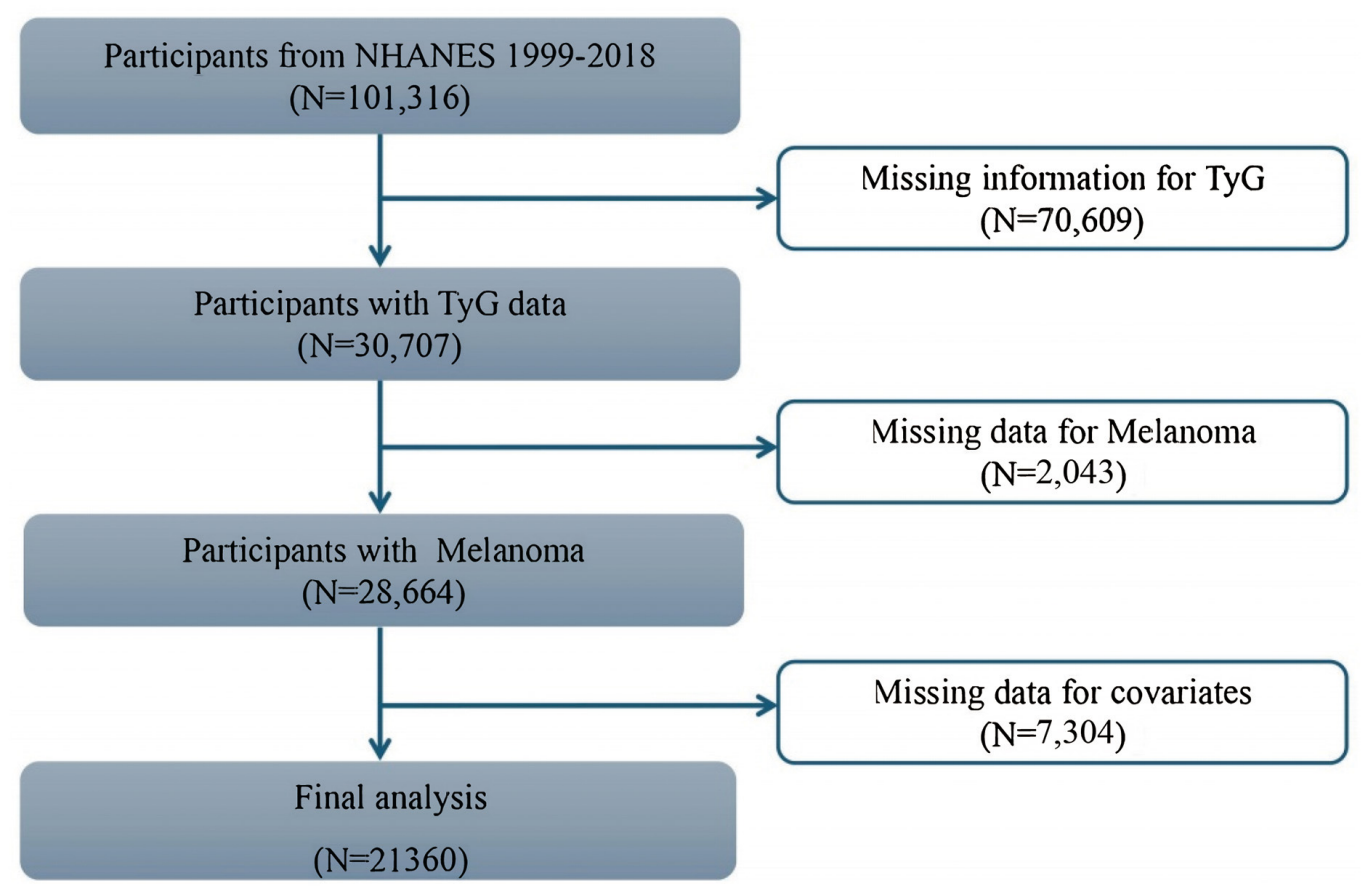
The flow diagram of participants selection.

This study utilized data from the National Health and Nutrition Examination Survey (NHANES), a large-scale, nationally representative survey conducted by the National Center for Health Statistics (NCHS) under the Centers for Disease Control and Prevention (CDC). The analysis incorporated data cycles spanning from 1999 to 2018. This study analyzed a total dataset of 101,316 individuals, a total of 70,609 participants with missing or invalid TyG data, 2,043 individuals with missing or invalid melanoma data, and 7,304 participants with missing or invalid other covariate data were excluded from the analysis. Ultimately, 21,360 participants aged 18 years or older were included in the final analysis, as summarized in the screening process illustrated in Figure 1. Detailed information about the survey methodology is available at http://www.cdc.gov/nchs/nhanes.htm. The study was authorized by the NCHS Research Ethics Review Board, and all participants provided written informed consent.

An external cohort was utilized to further describe the characteristics of the study population. An analysis of clinical data from a total of 475 patients diagnosed with acral melanoma who had undergone wide excision of the primary lesion between January 2006 and December 2021 was conducted. These patients were from three prominent tertiary hospitals in China: the Sun Yat-Sen University Cancer Center, the First Affiliated Hospital of Sun Yat-Sen University, and the Third Affiliated Hospital of Sun Yat-Sen University. In each hospital, an independent follow-up department conducted regular patient follow-ups, with the final follow-up date set at 30 June 2023, or the date of the patient's death.

This study was approved by the Medical Ethics Committee of Sun Yat-Sen University Cancer Center (approval no.: B2021-403-02). The patients in this manuscript have given written informed consent to publication of their case details.

### 2.2 Definition of the TyG index

The TyG index serves as a quantitative marker for insulin resistance, derived by combining fasting blood glucose and triglyceride levels. It is calculated using the formula: TyG = Ln [fasting triglycerides (mg/dL) × fasting glucose (mg/dL)/2] (10). Participants were stratified into 3 tertile groups (T1, T2, and T3) based on the TyG index values. The cutoff values for the tertile group were 5.65, 8.34, 8.89, and 13.40 in the study population. Specifically, T1 (the reference group) was defined as 5.65 < TyG ≤ 8.34, T2 as 8.34 < TyG ≤ 8.89, and T3 as 8.89 < TyG ≤ 13.40. These cutoff values are highly consistent with the tertile cutoff points obtained through windsorization (Supplementary Table 1).

### 2.3 Definition of melanoma

Participants who answered “Yes” to MCQ220 of the Medical Conditions Questionnaire (MCQ), “Have you ever been told by a doctor or health professional that you have a malignant tumor?” and “Melanoma” to MCQ230, “What type of cancer do you have?” were categorized as melanoma patients and included in the case group. Participants who answered “No” to MCQ220 were classified as non-melanoma and assigned to the control group.

### 2.4 Assessment of mortality

Mortality status was determined through probabilistic linkage with the National Death Index. Only participants with eligible follow-up time were included in the survival analysis. Participants who remained alive during the follow-up period, were lost to follow-up, or were administratively censored were coded as 0 (alive). Participants who died from any cause were coded as 1 (death). Survival time was calculated in months from the later of the NHANES interview or Mobile Examination Center visit date to the date of death or December 31, 2019 (administrative censoring), whichever occurred first.

### 2.5 Covariate

The demographic variables included in this study were age, sex (male, female), race (Mexican American, other Hispanic, non-Hispanic White, non-Hispanic Black, and other races), education, marital status, and income-poverty ratio. Additionally, personal history of smoking and alcohol consumption were included. Body mass index (BMI) measurements were taken at a mobile screening center. Blood samples were analyzed for metabolism-related markers, liver and kidney function markers, and other biochemical markers. Hypertension was diagnosed if systolic/diastolic blood pressure was ≥ 140/90 mmHg, if there was a self-reported physician diagnosis of hypertension, or if the participant was using hypertensive medications. Diabetes was diagnosed with an HbA1c ≥ 6.5%, a self-reported physician diagnosis of diabetes, or self-reported insulin use. Stroke was defined by a self-reported physician diagnosis of stroke. Dyslipidemia was defined by a self-reported physician diagnosis of high cholesterol or if the participant was currently using lipid-lowering medications. Chronic kidney disease (CKD) was defined as eGFR < 60 mL/min/1.73 m^2^. Participants were also asked, “Has a doctor or other health professional ever told you that you have heart failure or coronary heart disease?” Those who answered “Yes” to either question were classified as having heart failure or coronary heart disease.

### 2.6 Shapley additive explanations (SHAP)

To further interpret the RR model's prediction results, we visualized key features and their importance using the SHAP method. SHAP provides a way to explain individual predictions by computing the contribution of each feature to the prediction, helping to understand how features influence the model's output.

### 2.7 Statistical analysis

Survey sampling weights (WTMEC2YR), rescaled for pooled cycles to ensure national representativeness, were incorporated into the analysis. Strata (SDMVSTRA) and primary sampling units (SDMVPSU) were included in all statistical models to account for biases associated with the complex survey design. Consistent with the overall weighted analysis design, case weights were also used in all machine learning-related analyses to maintain sample representativeness.

Prior to analysis, missing data were addressed: variables with missing data exceeding 20% were excluded to minimize bias arising from incomplete data. Other variables with missing data of 20% (including age, gender, race, smoking status, alcohol consumption, BMI, etc.) or less were handled using multiple imputation by chained equations (MICE). Final report results are based on the imputed dataset with m = 5. Specific MICE settings are as follows: imputation was performed using the predictive mean matching method, with the predictive variable matrix including all variables relevant to the analysis. The iteration count was set to 10. To maintain consistency with the aforementioned weighted analysis design, survey design objects were constructed using the survey software package. These survey design variables, along with outcome variables (melanoma status and overall survival) and all analytical covariates, were incorporated into the interpolation model. To integrate results from five interpolated datasets while accounting for complex survey design, we fitted statistical models (weighted logistic/Cox regression) for each survey design object. Rubin's rules were applied to combine parameter estimates and their standard errors across interpolated datasets, ensuring robustness of final results. Additionally, sensitivity analyses using m =20 interpolated datasets were conducted to confirm robustness (Supplementary Table 2).

Continuous variables were represented by weighted means and standard deviations, while categorical variables were expressed as weighted percentages. Weighted *t*-tests and chi-square tests were employed to assess differences between continuous and categorical variables, respectively. Winsorization was adopted to conduct a sensitivity analysis on the TyG index. Weighted logistic regression analysis was performed to investigate whether TyG exposure could be considered a risk factor for melanoma, specifically examining the association between the TyG index and melanoma prevalence. The odds ratio (OR) and its 95% confidence interval (CI) were used to express the strength of the association. After assessment using the Schoenfeld residual test, weighted multivariate Cox proportional hazards regression was utilized to assess the association between TyG and all-cause mortality among patients with melanoma, with the strength of the association expressed through the hazard ratio (HR) and its 95% CI. To further examine this association, we developed three statistical models. Model 1 presented unadjusted results. Model 2 built upon Model 1 by adjusting for demographic factors including age, sex, and race. Finally, Model 3 was comprehensively adjusted for all potential covariates identified in the study. Restricted cubic splines (RCS) were employed to examine the dose-response relationship between the TyG index and all-cause mortality risk. Subgroup analyses were also conducted to validate the robustness of the results.

To enhance the prediction of melanoma, this study employed seven machine learning algorithms to develop a prediction model. In all machine learning models, we incorporated the survey weights (WTMEC2YR) as sample weights during model training to account for the complex survey design. However, due to the limitations of standard machine learning libraries, we could not directly incorporate strata and PSU information. The algorithms include Logistic Regression (LR), Extreme Gradient Boosting (XGBoost), Light Gradient Boosting Machine (LightGBM), Ridge Regression (RR), Decision Tree (DT), Elastic Net Regularization (EN), and Lasso Regression (Lasso). The data were randomly divided into training and validation sets, with 70% allocated to the training set and 30% to the validation set. Subsequent analyses were performed to comprehensively evaluate the model's actual clinical utility under conditions of extreme class imbalance, utilizing the precision-recall area under curve (PR-AUC), calibration plots/Brier score, and decision curve analysis (DCA), along with reporting the positive predictive value (PPV)/negative predictive value (NPV) at pre-specified clinical thresholds. A cohort of non-metastatic acral melanoma patients from an external institution was included for descriptive analysis. To optimize the predictive model and prevent overfitting, 10-fold cross-validation was conducted during model training. For the internal validation of the model, Receiver Operating Characteristic (ROC) curves were plotted to assess the discriminative performance, and the area under the curve (AUC) was calculated. The AUROC value was used as the primary statistical metric to assess the predictive performance of the model in the internal validation, with accuracy, sensitivity, and specificity also reported for comprehensive evaluation.

## 3 Results

### 3.1 Characteristics of participants

The cohort study initially comprised 21,360 participants, including 127 melanoma cases (weighted prevalence: 0.6%). Of the participants, 50 were male, representing a weighted percentage of 37.4%. Based on the diagnostic criteria for melanoma, the participants were stratified into melanoma and non-melanoma groups. Significant differences were observed between the two groups in terms of age, gender, median follow-up time, and race. In addition, laboratory analyses further revealed statistically significant differences between the two groups in levels of insulin, TyG index, triglyceride, potassium, globulin, uric acid, and total protein (Table 1). Additionally, the 25th percentile of the continuous TyG index was 8.19, the 50th percentile was 8.61, and the 75th percentile was 9.05, with a mean ± standard deviation of 8.62 ± 0.67. In the external data, there were 255 survivors and 220 deaths. The two groups showed significant differences in age, albumin, globulin, and survival time (Supplementary Table 3).

**Table 1:** Weighted baseline characteristics.

***n***	**Level**	**Overall**	**No**	**Yes**	***P***
		**21,360**	**21,233**	**127**	
Age (years)		48.25 (16.28)	48.15 (16.23)	65.10 (14.05)	< 0.001
Gender	Female	11,016 (50.60)	10,966 (50.70)	50 (37.40)	0.008
	Male	10,344 (49.40)	10,267 (49.30)	77 (62.60)	
Race	Mexican American	3,999 (8.70)	3,992 (8.80)	7 (1.60)	< 0.001
	Non-Hispanic Black	4,388 (11.10)	4,385 (11.20)	3 (0.80)	
	Non-Hispanic White	9,083 (67.20)	8,972 (66.90)	111 (95.00)	
	Other Hispanic	1,862 (5.90)	1,859 (6.00)	3 (0.80)	
	Other race	2,028 (7.10)	2,025 (7.10)	3 (1.80)	
BMI (kg/m^2^)		28.92 (6.76)	28.93 (6.77)	28.84 (5.77)	0.887
Insulin (μU/mL)		13.67 (14.83)	13.63 (14.39)	19.72 (43.23)	< 0.001
Glucose (mg/dL)		107.69 (30.78)	107.67 (30.80)	110.83 (27.63)	0.328
HbA1c (%)		5.70 (0.92)	5.70 (0.92)	5.78 (0.76)	0.408
TyG		8.65 (0.67)	8.65 (0.67)	8.79 (0.62)	0.029
Triglyceride (mg/dL)		134.00 (118.85)	133.84 (115.62)	160.64 (331.12)	0.012
Total cholesterol (mg/dL)		5.05 (1.08)	5.05 (1.07)	5.03 (1.50)	0.809
Total calcium (mg/dL)		9.36 (0.36)	9.36 (0.36)	9.36 (0.37)	0.995
Potassium (mmol/L)		4.03 (0.34)	4.03 (0.34)	4.10 (0.39)	0.034
Phosphorus (mmol/L)		1.17 (0.18)	1.17 (0.18)	1.14 (0.21)	0.060
Albumin (g/dL)		4.20 (0.35)	4.20 (0.35)	4.17 (0.37)	0.332
Globulin (g/dL)		3.00 (0.43)	3.01 (0.43)	2.90 (0.43)	0.012
Uric acid (μmol/L)		323.78 (83.67)	323.59 (83.67)	355.08 (80.20)	< 0.001
Total bilirubin (μmol/L)		11.84 (5.32)	11.84 (5.32)	11.82 (5.35)	0.967
Total protein (g/dL)		7.21 (0.47)	7.21 (0.47)	7.07 (0.45)	0.002
Serum creatinine (mg/dL)		0.87 (0.38)	0.87 (0.38)	1.07 (0.69)	< 0.001
Blood urea nitrogen (mg/dL)		13.35 (5.16)	13.33 (5.15)	16.17 (5.74)	< 0.001
Iron (μg/dL)		88.93 (36.77)	88.93 (36.80)	88.71 (32.46)	0.946
Median follow-up time (months)		119 (65–178)	115 (63–172)	76 (46–136)	< 0.001
Diabetes	No	18,547 (91.60)	18,447 (91.70)	100 (85.20)	0.062
	Yes	2,420 (8.40)	2,399 (8.30)	21 (14.80)	
Hypertension	No	14,287 (70.60)	14,230 (70.80)	57 (43.10)	< 0.001
	Yes	7,073 (29.40)	7,003 (29.20)	70 (56.90)	
Coronary heart	No	20,591 (96.90)	20,476 (97.00)	115 (92.00)	0.001
	Yes	769 (3.10)	757 (3.00)	12 (8.00)	
Heart failure	No	20,749 (98.00)	20,629 (98.00)	120 (95.10)	0.126
	Yes	611 (2.00)	604 (2.00)	7 (4.90)	
Osmotic pressure (mOsm/kg)		278.33 (5.03)	278.32 (5.02)	279.95 (5.12)	0.001
Time		118.96 (68.30)	119.11 (68.30)	94.47 (66.61)	< 0.001

Continuous variables are expressed as mean (standard deviation), while categorical variables are expressed as frequency (%).

### 3.2 Mortality analysis

After stratification based on the TyG index, the T3 group had the highest mortality rate (18.64%), followed by T2 (14.13%) and T1 (7.9%) (Supplementary Table 4). KM curves indicate that cumulative survival rates for all three patient groups gradually declined over the observation period (Supplementary Figure 1). Among them, the T3 group exhibited the steepest decline in cumulative survival rates, with the lowest cumulative survival rates at all time points. The T2 group demonstrated an intermediate rate of decline and survival levels. The T1 group showed the flattest survival curve.

### 3.3 Analysis of the association between TyG and melanoma by weighted logistic regression

To investigate the association between the TyG index and the prevalence of melanoma, we performed a multivariate weighted logistic regression analysis to calculate the OR with corresponding 95% confidence intervals (Table 2). In Model 1, no adjustments were made for confounding factors. Model 2 incorporated adjustments for demographic variables including age, sex, and race. Model 3 included comprehensive adjustment for all potential confounding factors. The results indicated no statistically significant overall association between the TyG index and melanoma risk. Although the OR for the T3 was significantly elevated compared to the T1 in Model 1 (OR = 1.885; 95% CI: 1.066–3.333; *P* = 0.031), this association lost statistical significance after further adjustment for confounding factors.

**Table 2:** Multivariate weighted logistic regression analysis.

**TyG**	**Model 1**	***P* value**	**Model 2**	***P* value**	**Model 3**	***P* value**
	**OR (95%CI)**		**OR (95%CI)**		**OR (95%CI)**	
T1	Reference					
T2	1.486 (0.8–2.761)	0.212	1.004 (0.525–1.92)	0.99	0.999 (0.994–1.005)	0.826
T3	1.885 (1.066–3.333)	0.031	1.096 (0.605–1.984)	0.764	0.998 (0.988–1.008)	0.694

### 3.4 Positive correlation between TyG exposure levels and all-cause mortality in melanoma patients

To evaluate the potential of the TyG index as a prognostic factor in patients with melanoma, the Schoenfeld residual test was performed with a resultant *P* > 0.05 (Supplementary Figure 2); subsequently, a weighted cox regression model was applied for survival analysis. Consecutive TyG index values were categorized into their tertiles for the Cox regression analysis (Table 3). The analysis initially suggested a potential positive association between TyG index levels and all-cause mortality among melanoma patients. In Model 2, the HR for the T3 was significantly elevated compared to the T1 (HR = 1.329, 95% CI: 1.201–1.458, *P* < 0.05), indicating a 33% higher risk of mortality. However, this association did not remain statistically significant after full adjustment for all confounding factors in the final model.

**Table 3:** Multivariate weighted cox regression analysis.

**TyG**	**Model 1**	***P* value**	**Model 2**	***P* value**	**Model 3**	***P* value**
	**HR (95%CI)**		**HR (95%CI)**		**HR (95%CI)**	
T1	Reference					
T2	1.707 (1.576–1.838)	< 0.01	1.011 (0.892–1.130)	0.858	0.987 (0.860–1.114)	0.841
T3	2.601 (2.459–2.744)	< 0.01	1.329 (1.201–1.458)	< 0.010	1.100 (0.937–1.264)	0.252

### 3.5 RCS analysis of TyG index and all-cause mortality

**Figure 2:**
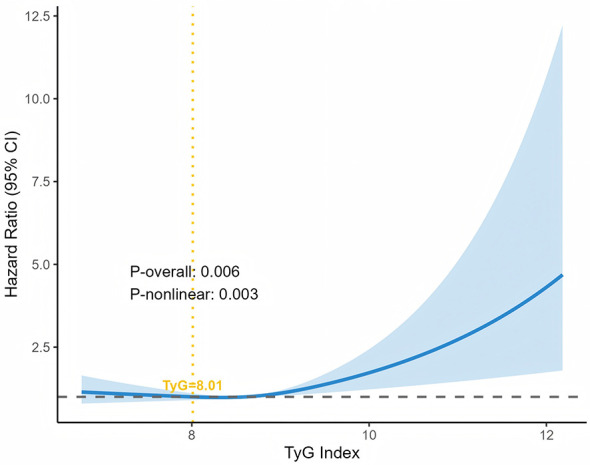
Restricted cubic spline for the relationship between TyG index and melanoma in different models.

To examine the non-linear association between TyG index and all-cause mortality among melanoma patients, we performed RCS analysis. As shown in Figure 2, after adjustment for all covariates, the model confirmed these findings, showing significantly elevated all-cause mortality risk at TyG index levels >8.01 (*P*-overall = 0.006; *P*-non-linear = 0.003). Knots were placed at the 5th, 50th, and 95th percentiles of the TyG index distribution. These results indicate a non-linear association between the TyG index and all-cause mortality, with positive correlations observed above specific thresholds.

### 3.6 Subgroup analysis

Following the initial identification of a potential link, we aimed to investigate whether this trend persisted across population subgroups. To address these questions, we conducted comprehensive subgroup and interaction analyses (Table 4). Stratified analyses suggested that among melanoma patients aged ≤ 65 years, the TyG index tended to show a significant positive association with all-cause mortality (HR = 1.89, 95% CI: 1.71–2.10, *P* < 0.001). Regarding sex differences, the interaction analysis revealed a significant effect modification, with the TyG index-all-cause mortality association appearing significantly more pronounced in females (HR = 2.09, 95% CI: 1.89–2.32, *P* < 0.001) than in males. Additionally, our analyses indicated that metabolic dysregulation markers and comorbid conditions significantly modified this association. Specifically, hyperuricemia, elevated blood urea nitrogen levels, diabetes mellitus, hypertension, coronary artery disease, and congestive heart failure were all associated with significantly increased all-cause mortality risk in melanoma patients with elevated TyG index levels.

**Table 4:** Subgroup analysis.

**Variable**	**Count**	**Percent**	**HR (95% CI)**	***P***	***P* for interaction**
Overall	21,360	100.0	1.69 (1.58–1.81)	< 0.001	
**Age**					< 0.001
< =65	17,155	80.3	1.89 (1.71–2.10)	< 0.001	
>65	4,205	19.7	1.03 (0.94–1.13)	0.523	
**Gender**					< 0.001
Female	11,016	51.6	2.09 (1.89–2.32)	< 0.001	
Male	10,344	48.4	1.42 (1.30–1.55)	< 0.001	
**Race**					0.494
Mexican American	3,999	18.7	1.73 (1.49–2.00)	< 0.001	
Non-Hispanic Black	4,388	20.5	1.82 (1.59–2.08)	< 0.001	
Non-Hispanic White	9,083	42.5	1.70 (1.56–1.85)	< 0.001	
Other Hispanic	1,862	8.7	1.68 (1.35–2.08)	< 0.001	
Other race	2,028	9.5	1.95 (1.39–2.74)	< 0.001	
**Glucose**					0.107
Q1	5,741	26.9	1.44 (1.15–1.82)	0.002	
Q2	5,173	24.2	1.27 (1.07–1.50)	0.006	
Q3	5,177	24.2	1.33 (1.15–1.54)	< 0.001	
Q4	5,269	24.7	1.15 (1.04–1.27)	0.008	
**Globulin**					0.359
Q1	6,346	29.7	1.73 (1.53–1.95)	< 0.001	
Q2	5,949	27.9	1.65 (1.44–1.89)	< 0.001	
Q3	4,781	22.4	1.71 (1.51–1.94)	< 0.001	
Q4	4,284	20.1	1.58 (1.40–1.78)	< 0.001	
**Uric acid**					< 0.001
Q1	5,591	26.2	2.01 (1.76–2.29)	< 0.001	
Q2	5,186	24.3	1.73 (1.51–1.99)	< 0.001	
Q3	5,248	24.6	1.68 (1.46–1.92)	< 0.001	
Q4	5,335	25.0	1.26 (1.10–1.44)	0.001	
**Total protein**					0.102
Q1	6,291	29.5	1.61 (1.44–1.79)	< 0.001	
Q2	5,402	25.3	1.64 (1.43–1.88)	< 0.001	
Q3	4,755	22.3	1.76 (1.55–1.99)	< 0.001	
Q4	4,912	23.0	1.83 (1.64–2.04)	< 0.001	
**Osmotic_pressure**					0.274
Q1	5,983	28.0	1.45 (1.28–1.63)	< 0.001	
Q2	5,301	24.8	1.63 (1.42–1.88)	< 0.001	
Q3	4,778	22.4	1.70 (1.49–1.95)	< 0.001	
Q4	5,298	24.8	1.58 (1.43–1.75)	< 0.001	
**Phosphorus**					0.987
Q1	6,309	29.5	1.65 (1.47–1.86)	< 0.001	
Q2	4,972	23.3	1.71 (1.47–1.98)	< 0.001	
Q3	5,654	26.5	1.77 (1.56–2.00)	< 0.001	
Q4	4,425	20.7	1.64 (1.44–1.88)	< 0.001	
Q1	6,420	30.1	1.83 (1.56–2.14)	< 0.001	
Q2	4,598	21.5	1.73 (1.50–2.00)	< 0.001	
Q3	5,960	27.9	1.51 (1.35–1.69)	< 0.001	
Q4	4,382	20.5	1.51 (1.35–1.68)	< 0.001	
**Blood_urea_nitrogen**					0.002
Q1	6,626	31.0	1.91 (1.62–2.26)	< 0.001	
Q2	4,115	19.3	1.64 (1.37–1.96)	< 0.001	
Q3	6,223	29.1	1.57 (1.40–1.77)	< 0.001	
Q4	4,396	20.6	1.40 (1.27–1.56)	< 0.001	
**Diabetes**					< 0.001
No	18,547	88.5	1.57 (1.44–1.72)	< 0.001	
Yes	2,420	11.5	1.07 (0.96–1.21)	0.227	
**Hypertension**					< 0.001
No	14,287	66.9	1.75 (1.59–1.93)	< 0.001	
Yes	7,073	33.1	1.23 (1.12–1.35)	< 0.001	
**Corpore heart**					< 0.001
No	20,591	96.4	1.70 (1.58–1.82)	< 0.001	
Yes	769	3.6	0.96 (0.77–1.20)	0.729	
**Heart failure**					< 0.001
No	20,749	97.1	1.69 (1.57–1.81)	< 0.001	
Yes	611	2.9	0.85 (0.67–1.08)	0.185	

### 3.7 Machine learning (ML) algorithms for predicting melanoma

**Figure 3:**
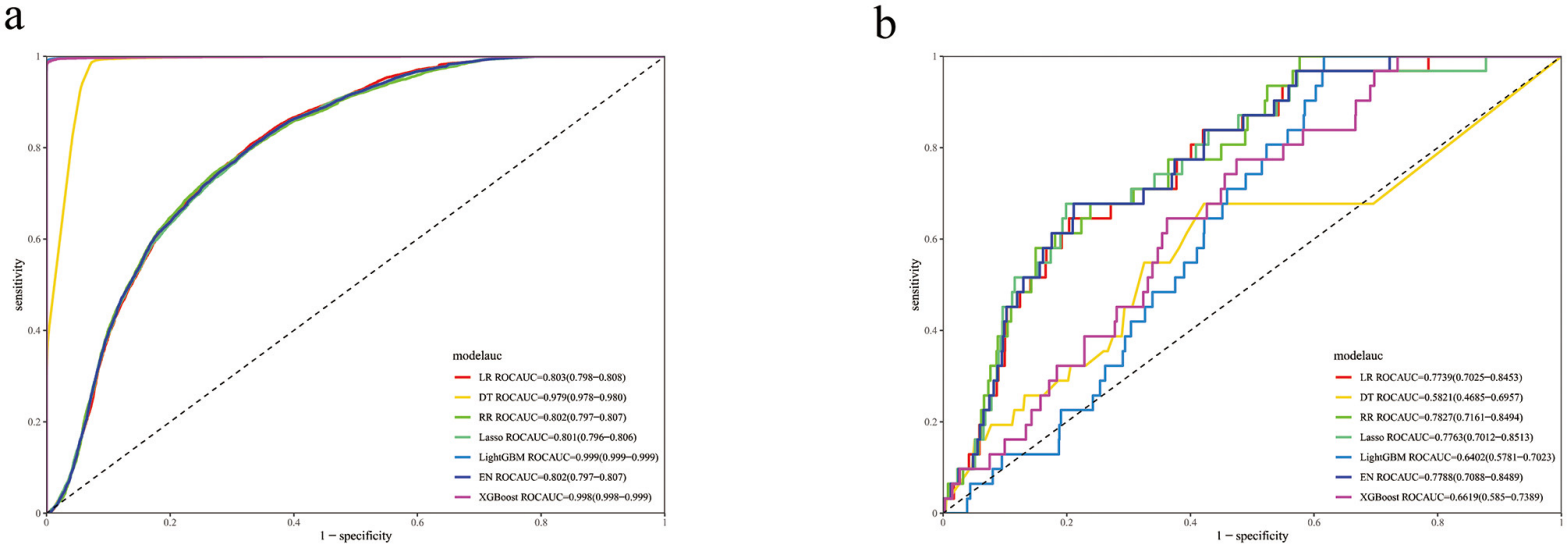
ROC curves for seven models. **(a)** ROC in the training set. **(b)** ROC in the validation set.

As shown in Figure 3, the ROC analysis revealed significant performance differences between training and validation sets. While the LightGBM, XGBoost, and Decision Tree (DT) models achieved exceptionally high AUROC values in the training set (LightGBM: 0.999; XGBoost: 0.999; DT: 0.999), their validation performance dropped markedly (LightGBM: 0.82; XGBoost: 0.82; DT: 0.61), indicating severe overfitting. In contrast, the RR model maintained consistent performance between training and validation phases, exhibiting excellent generalizability without evidence of overfitting. Similar stability was observed for LR and EN, but their predictive power (LR AUROC = 0.84; EN AUROC = 0.82) was lower than RR (Table 5).

**Table 5:** Comparison of discrimination characteristics among seven machine learning models.

**Model name**	**Accuracy**	**Sensitivity**	**Specificity**	**Precision**	**F means**	**ROCAUC**
LR	0.727	0.865	0.726	0.018	0.036	0.844
DT	0.979	0.108	0.984	0.039	0.057	0.607
RR	0.696	0.865	0.695	0.017	0.032	0.85
Lasso	0.705	0.865	0.704	0.017	0.033	0.848
LightGBM	0.991	0.000	0.997	0.000	0.000	0.818
EN	0.665	0.800	0.665	0.013	0.025	0.825
XGBoost	0.987	0.027	0.993	0.021	0.024	0.819
LR	0.797	0.873	0.721	0.758	0.811	0.856
DT	0.994	0.995	0.992	0.992	0.994	0.999
RR	0.793	0.893	0.693	0.744	0.812	0.855
Lasso	0.795	0.888	0.701	0.748	0.812	0.856
LightGBM	0.994	0.989	0.998	0.998	0.994	1.000
EN	0.791	0.908	0.673	0.735	0.813	0.863
XGBoost	0.993	0.991	0.994	0.994	0.993	0.999

LR, Logistic Regression; DT, Decision Tree; RR, Ridge Regression; Lasso, Lasso Regression; LightGBM, Light Gradient Boosting Machine; EN, Elastic Net Regularization; XGBoost, Extreme Gradient Boosting.

### 3.8 Performance validation of models on class imbalanced data

This study constructed tree models (XGBoost, LightGBM, DT) and linear models (LR, EN, Lasso, RR) for extremely imbalanced data. Model performance was evaluated using PR-AUC, calibration curves, DCA, and quantitative indicators (Brier score, PPV, NPV) (Supplementary Tables 5, 6 and Supplementary Figures 3–5). For extremely imbalanced melanoma data, tree models and linear models performed well in the training set, with positive DCA net benefits; tree models showed better PPV/NPV (PPV: 0.9305–0.9964, NPV: 0.9862–0.9931) than linear models (PPV: 0.7181–0.7228, NPV: 0.7459–0.7519). However, in the test set, all models exhibited DCA net benefits approaching 0 and PPV values close to 0. Supported by PR-AUC and calibration curve analyses.

### 3.9 SHAP value

**Figure 4:**
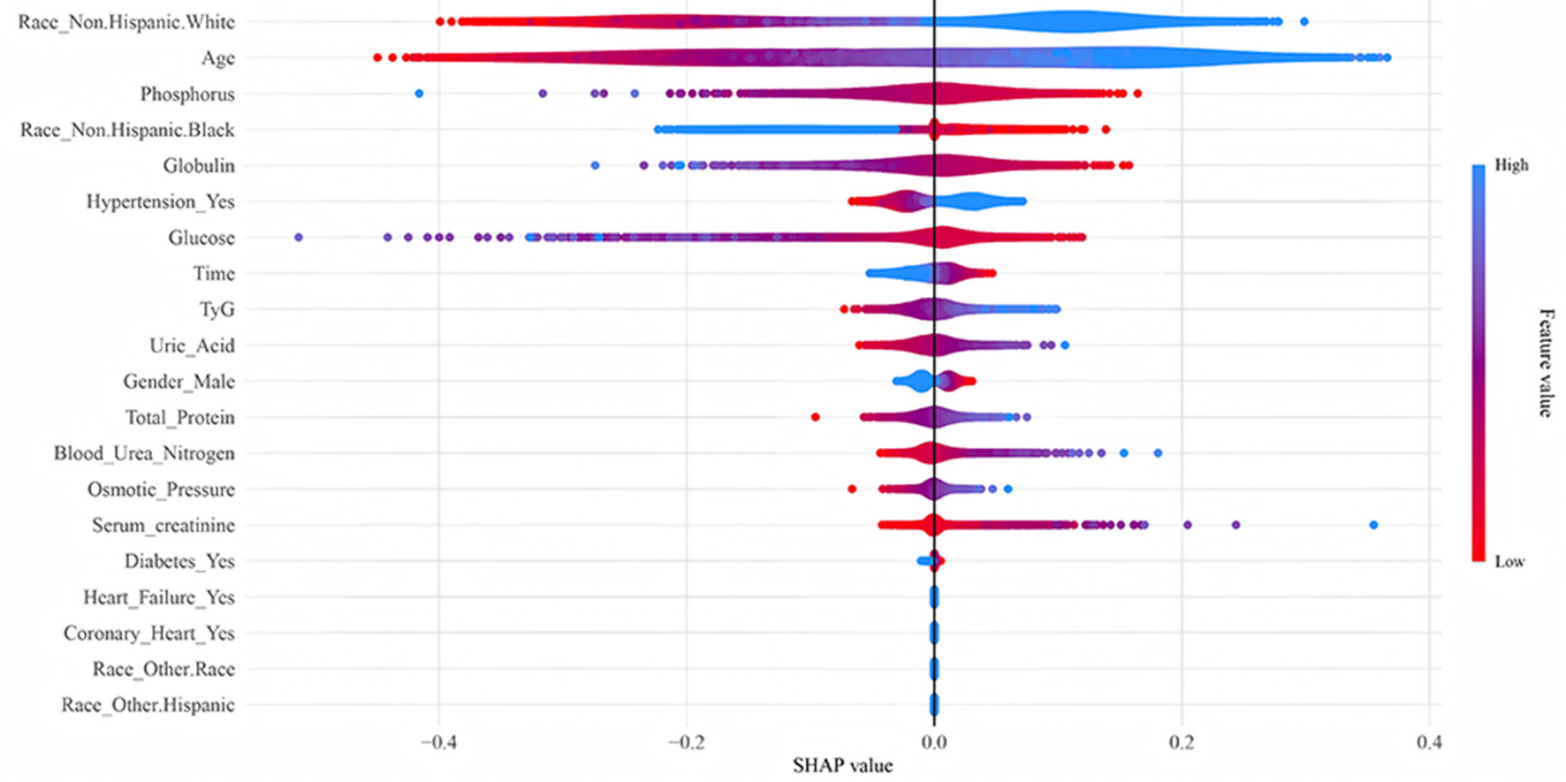
Summary plot of SHAP values for the model constructed by RR algorithm.

As illustrated in the SHAP summary plot (Figure 4), we visualized key features to the RR model's predictions. Each point on the plot represents a specific sample, with its horizontal position reflecting the influence of the corresponding feature on the predicted probability. The magnitude of the feature value is indicated by color, where red represents a lower feature value and blue represents a higher feature value. The SHAP value quantitatively measures the impact of each feature on the predicted outcome, with a positive SHAP value indicating a positive correlation between the feature value and melanoma prevalence, and a negative SHAP value indicating a negative correlation. Race was identified as a significant predictor of melanoma risk; higher SHAP values were observed at white race, indicating an increased risk of melanoma. Furthermore, metabolic metrics, including age, phosphorus, globulin, blood glucose, and the TyG index, were found to significantly influence model predictions. These findings align with previous research highlighting the association between melanoma and metabolic disorders.

## 4 Discussion

This study utilized NHANES data (1999–2018) from 21,360 participants to investigate the relationship between the TyG index and melanoma risk. The results revealed a “U-shaped” dose-response relationship between the TyG index and all-cause mortality, while the positive association disappeared after full adjustment for all covariates in traditional regression models. Importantly, the RR machine learning model demonstrated relatively optimal performance. This model integrates the TyG index and other key variables, offering some reference for melanoma risk assessment.

The TyG index is a critical metabolic marker that reflects insulin resistance (10). Numerous studies have established a strong link between insulin resistance and the development of various malignant tumors, including colorectal cancer (18), breast cancer (19), and liver cancer (20). While the hyperinsulinemic-euglycemic clamp technique is considered the “gold standard” for diagnosing insulin resistance, its high cost and complexity limit its widespread application in routine clinical practice (21). In contrast, the TyG index, which can be calculated through routine testing, is simple, cost-effective, and regarded as one of the alternative indicators for assessing insulin resistance. The mechanisms through which the TyG index and insulin resistance contribute to melanoma development and progression are likely associated with the pro-proliferative effects of insulin, lipid metabolism disturbances, and systemic low-grade inflammation (22). Studies suggest that elevated insulin levels can promote abnormal melanocyte proliferation and accelerate tumorigenesis by activating insulin receptors and the downstream PI3K/Akt/mTOR pathways (23). Moreover, an increased TyG index typically corresponds with higher levels of fasting glucose and triglycerides. A cross-sectional study found a significant positive correlation between the risk of melanoma metastasis and fasting glucose and triglyceride concentrations (8). Beyond metabolic disturbances, the role of insulin resistance in promoting melanoma is closely linked to chronic inflammation (24). Insulin resistance induces adipocyte dysfunction, leading to an increased infiltration of M1-type pro-inflammatory macrophages in adipose tissue, prompting the release of inflammatory factors, including tumor necrosis factor-alpha, interleukin-6, and interleukin-1beta (25, 26). In a study by Molinelli et al. (27), tumor necrosis factor-alpha expression was significantly elevated in melanoma samples from peripheral tissues, further supporting the crucial role of tumor necrosis factor-alpha in local melanoma growth and invasion. Thus, the TyG index not only serves as a simple assessment tool for insulin resistance but also provides a valuable entry point for investigating the mechanisms of insulin resistance in melanoma. Further in-depth mechanistic studies are needed to clarify the causal relationship between the TyG index and melanoma development and to explore the potential benefits of intervening in insulin resistance for melanoma prevention.

Predictive tools are widely used in medical research and clinical practice for disease diagnosis and prognosis. Scoppola et al. (28) developed a column-line diagram to assess melanoma risk based on parameters such as body mass index and fasting blood glucose. However, traditional column-line diagrams are primarily designed for linear and relatively simple prediction scenarios, which limit their ability to capture complex patterns and non-linear relationships. In contrast, machine learning techniques excel at handling multidimensional features and non-linear relationships, which improves the accuracy of predictive models. Machine learning models have been widely applied in cancer risk prediction across various cancer types. For instance, Xu et al. (29) compared several algorithms and found that the XGBoost model performed best in predicting bladder cancer risk. In our study, after constructing and evaluating seven mainstream machine learning algorithms, we found that the RR model demonstrated the best performance in predicting melanoma risk. Given the low prevalence of melanoma, further analysis revealed that the model's predictive capability is limited. Nevertheless, based on the findings of this study, it may still provide some reference value for subsequent research.

Our subgroup analysis indicated that metabolic disorder-related indicators and associated chronic diseases such as elevated uric acid, blood urea nitrogen, diabetes mellitus, hypertension, coronary heart disease, and heart failure significantly increased the risk of mortality in melanoma patients. These findings suggest that the TyG index, as a marker of insulin resistance, is often accompanied by these comorbidities, and their collective presence likely amplifies the overall risk of melanoma. Notably, the chronic inflammatory state and metabolic derangements characteristic of these conditions (13, 30), may foster a tumor-promoting microenvironment, further contributing to the development and progression of melanoma alongside the direct effects of insulin resistance captured by the TyG index.

Beyond the TyG index, our SHAP value analysis highlighted race, age, and serum phosphorus levels as the most important factors in the RR model's predictions. These findings align with known risk factors for melanoma, and our subgroup analyses provide further context for how certain variables might interact with or moderate the impact of the TyG index on melanoma prevalence. The TyG index showed a significantly positive association with all-cause mortality in patients ≤ 65 years and female patients. This suggests that age and sex may modulate the relationship between insulin resistance, as indicated by the TyG index, and mortality among patients with melanoma. The enhanced risk observed in younger patients may reflect more dynamic metabolic responses to insulin resistance that could potentiate melanoma progression. Similarly, hormonal differences in females could influence how the TyG index affects melanoma.

The SHAP values further indicated that age, blood glucose, phosphorus, and the TyG index itself significantly influenced model predictions. Given that the TyG index is derived from fasting glucose and triglycerides, its interplay with other metabolic markers is crucial. Higher blood glucose levels (a component of the TyG index) directly correlated with increased melanoma risk in the SHAP analysis. Similarly, we found an abnormal decline in serum phosphorus levels to be associated with elevated melanoma risk, potentially through its impact on vitamin D function (31). While the exact mechanistic link between low phosphorus and the TyG index needs further exploration, both are indicators of metabolic health, and their combined influence could exacerbate the risk of melanoma development or progression.

Although this study analyzed the association between the TyG index and melanoma using the large-scale NHANES database and employed machine learning models to explore potential correlations, several limitations remain. First, the low prevalence of melanoma in this cohort led to model overfitting. Then, the influence of unidentified confounding factors cannot be ruled out. Melanoma status in NHANES is self-reported, potentially leading to misclassification. Additionally, while we applied sample weights during model training, the inability to incorporate strata and PSU may lead to underestimation of variance in the machine learning models. Finally, given its limited clinical utility, further expansion of the sample size and multicenter validation are required to enhance its feasibility for clinical translation.

In summary, the TyG index exhibits a U-shaped association with all-cause mortality in patients with melanoma. Its significant impact on melanoma risk stratification was demonstrated in SHAP analysis, and its interactions with key variables like age, gender, and metabolic indicators in subgroup analysis fully highlight its clinical significance. The collective influence of the TyG index and these metabolically linked covariates, rather than isolated factors, likely plays a more comprehensive role in determining melanoma risk.

## 5 Conclusions

We found that the TyG index was associated with all-cause mortality in a U-shaped manner. In addition, the RR model exhibited superior predictive performance in the internal validation of melanoma risk assessment. This study provides valuable insights for the early identification of individuals at high risk for melanoma, the optimization of risk stratification, and the reduction of screening costs, particularly in clinical practice and large-scale epidemiological studies.

## Data Availability

The original contributions presented in the study are included in the article/Supplementary material, further inquiries can be directed to the corresponding authors.

## Supplementary material

Supplementary Figure 1 Survey-weighted TyG tertile cumulative survival curve.

Supplementary Figure 2 Schoenfeld residuals plot for proportional hazards assumption.

Supplementary Figure 3 Comparison of precision-recall curve area under the curve (PR-AUC) for training **(A)** and test **(B)** sets across models.

Supplementary Figure 4 Comparison of calibration curves for training **(A)** and testing **(B)** sets across models.

Supplementary Figure 5 Decision Curve Analysis (DCA) comparison of training **(A)** and testing **(B)** sets across models.

Supplementary Table 1 Sensitivity analysis findings of winsorization on the TyG index.

Supplementary Table 2 Sensitivity analysis of multiple imputation (m = 20) for TyG index and melanoma.

Supplementary Table 3 Baseline characteristics of the external cohort.

Supplementary Table 4 Number of deaths (or events) by TyG index stratification levels.

Supplementary Table 5 Comparison of calibration indicators (Brier Score) of each model in training and test sets.

Supplementary Table 6 PPV and NPV of each model at preset threshold (0.5).
