# Supplementary figures and images for: Machine learning-based association analysis of triglyceride-glucose index with melanoma prevalence and all-cause mortality: insights from cross-sectional NHANES 1999–2018 data and an external hospital-based dataset
Source: Front Nutr. 2026 Mar 18;13:1726865. doi: 10.3389/fnut.2026.1726865 (PMC13038597; doi:10.3389/fnut.2026.1726865)

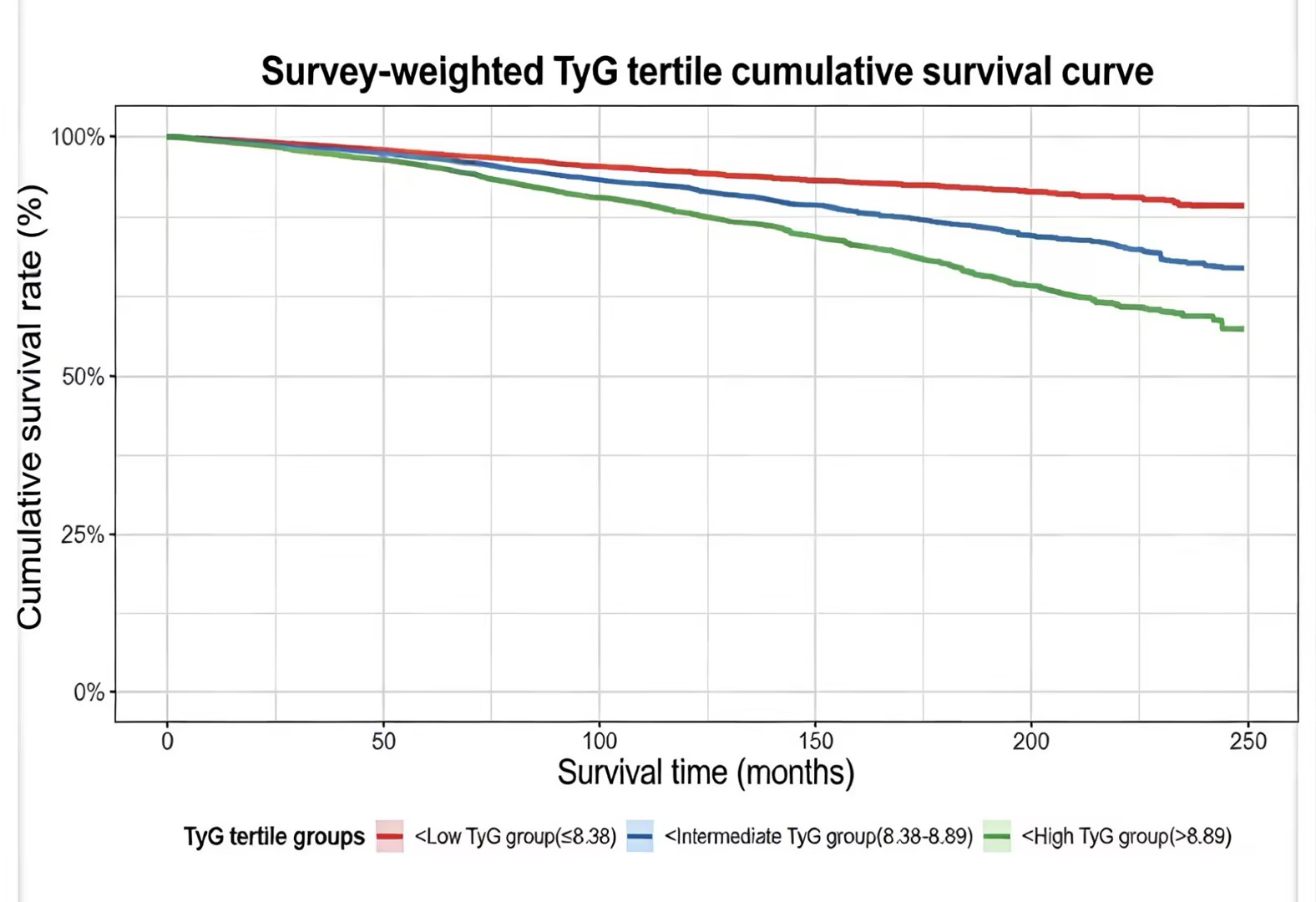

Supplement: Supplementary Figure 1 — Survey-weighted TyG tertile cumulative survival curve. [file Image_1.jpeg]

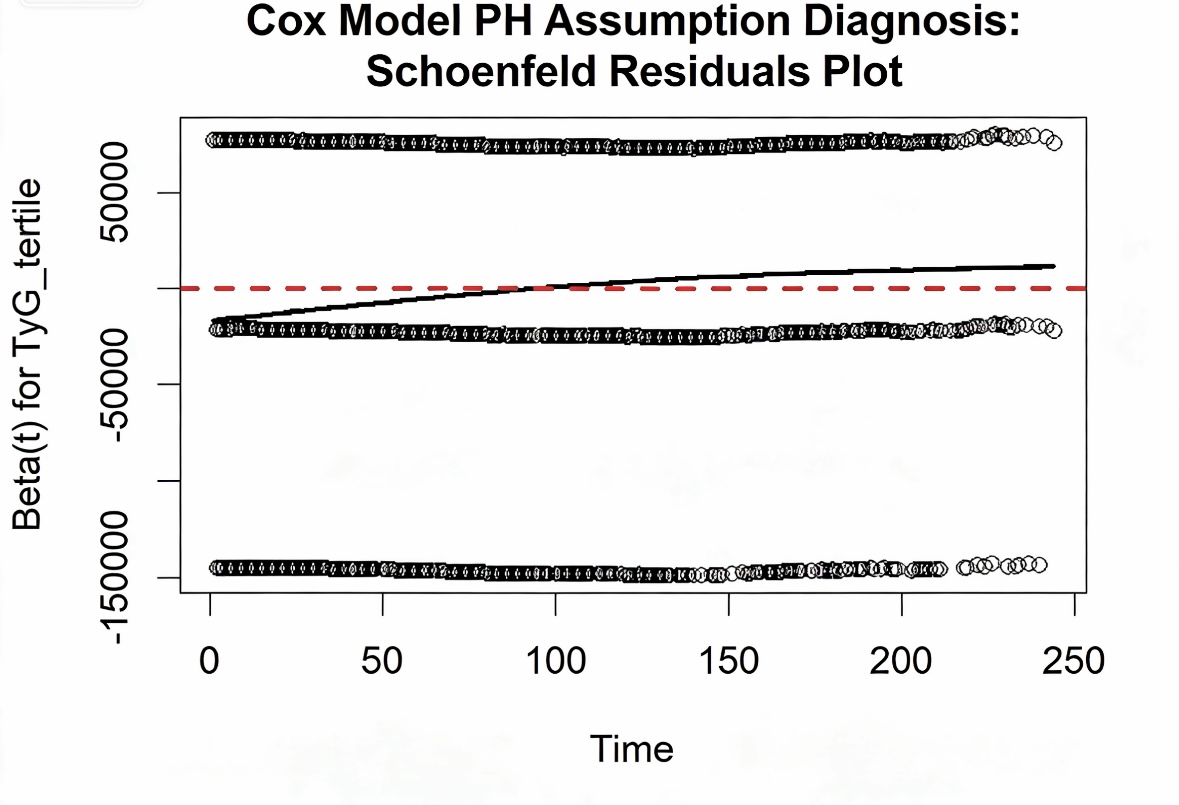

Supplement: Supplementary Figure 2 — Schoenfeld residuals plot for proportional hazards assumption. [file Image_2.jpeg]

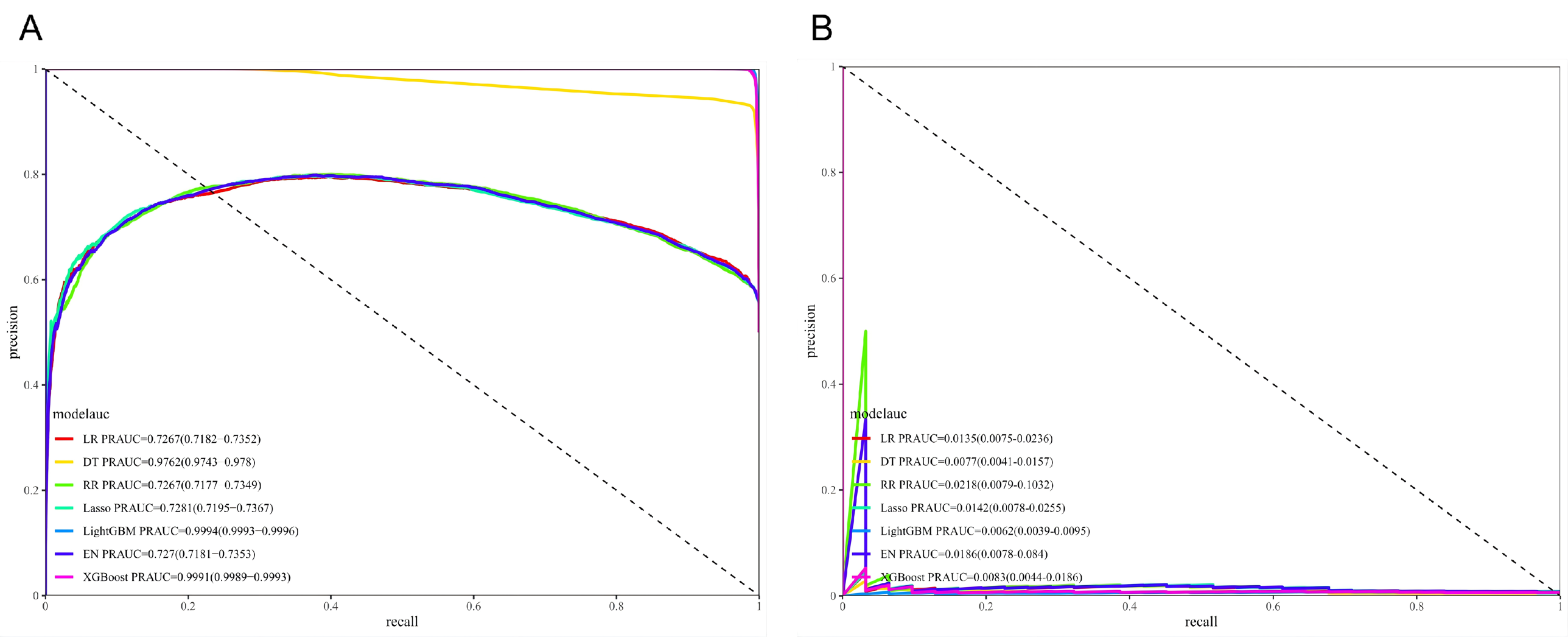

Supplement: Supplementary Figure 3 — Comparison of precision-recall curve area under the curve (PR-AUC) for training (A) and test (B) sets across models. [file Image_3.jpeg]

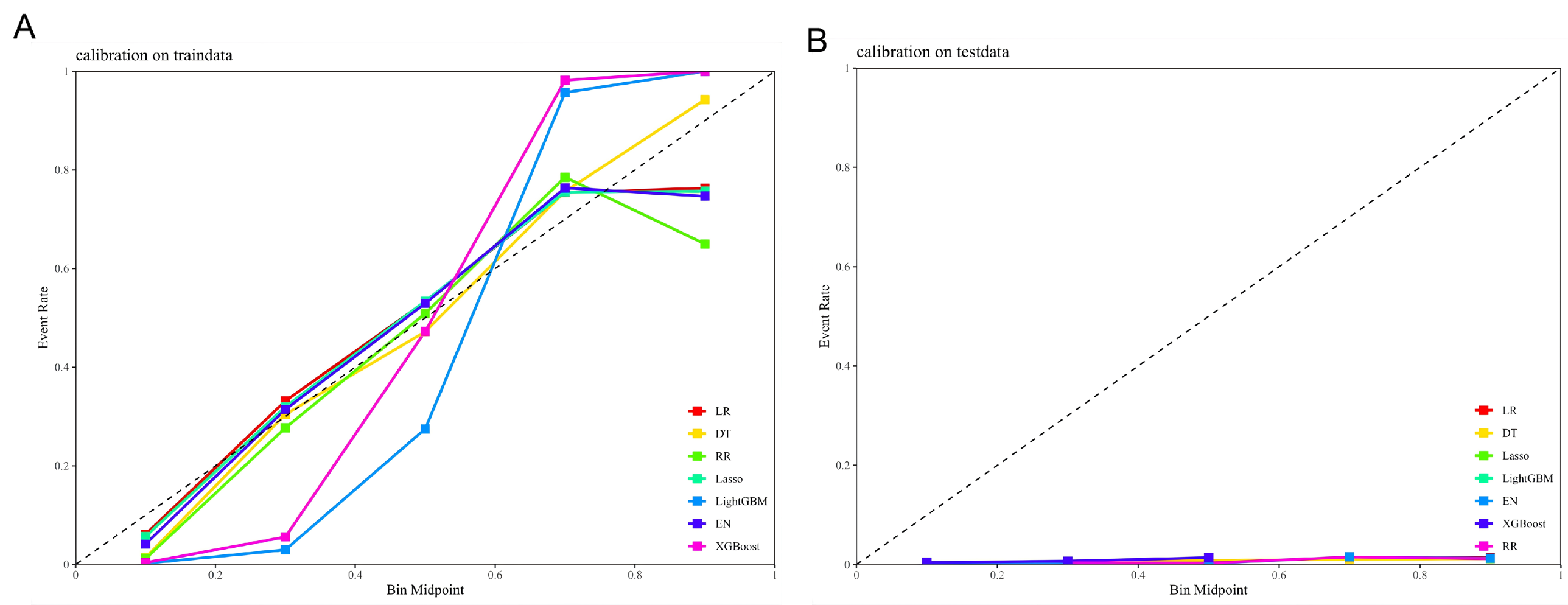

Supplement: Supplementary Figure 4 — Comparison of calibration curves for training (A) and testing (B) sets across models. [file Image_4.jpeg]

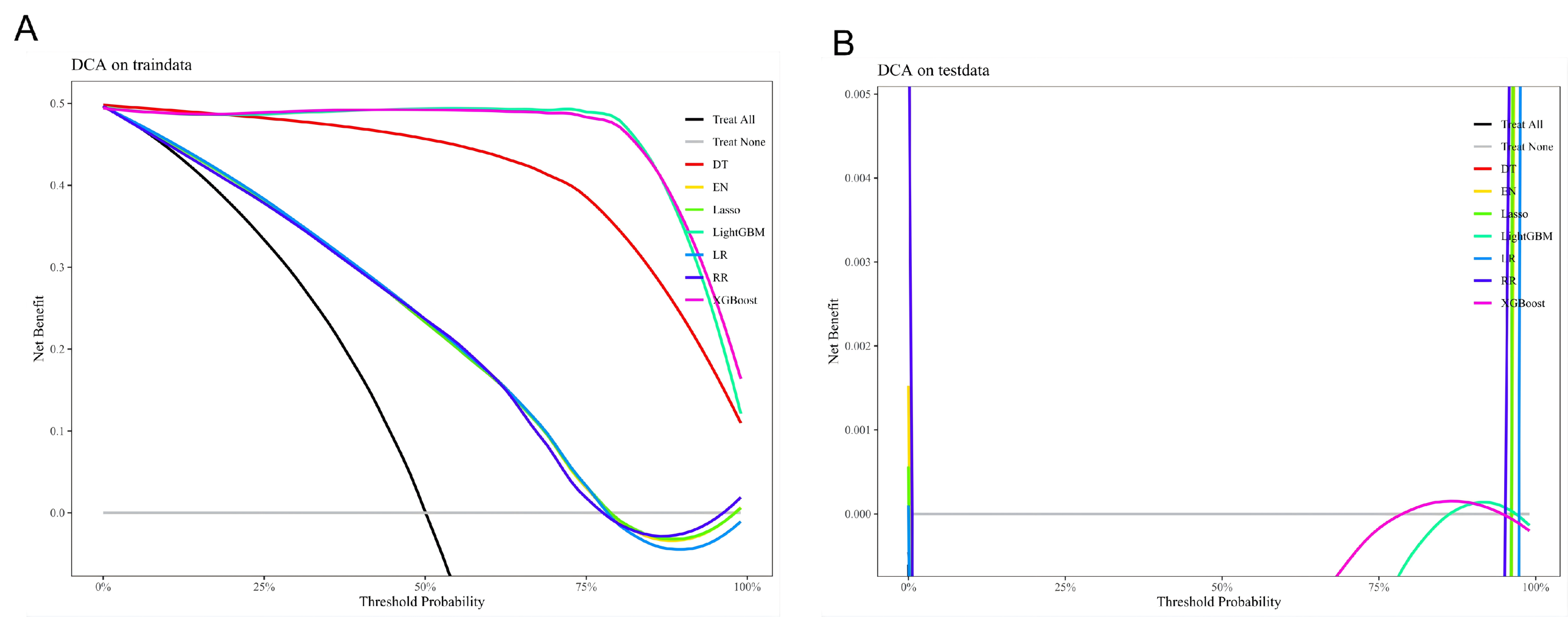

Supplement: Supplementary Figure 5 — Decision Curve Analysis (DCA) comparison of training (A) and testing (B) sets across models. [file Image_5.jpeg]
