# Supplementary material for: Machine learning-based association analysis of triglyceride-glucose index with melanoma prevalence and all-cause mortality: insights from cross-sectional NHANES 1999–2018 data and an external hospital-based dataset
Source: Front Nutr. 2026 Mar 18;13:1726865. doi: 10.3389/fnut.2026.1726865 (PMC13038597; doi:10.3389/fnut.2026.1726865)
Supplement: Supplementary Table 1 — Sensitivity analysis findings of winsorization on the TyG index. [file Table_1.docx]

**Supplementary Table 1 Sensitivity analysis findings of winsorization on the TyG index**

| Indicators | Results |
| --- | --- |
| Proportion | 1%/99% |
| TyG Range | 7.2654-10.6324 |
| TyG Tertile Cut-off 1 | 8.3360 (33.3%) |
| TyG Tertile Cut-off 2 | 8.8901 (66.7%) |
