# Supplementary material for: Machine learning-based association analysis of triglyceride-glucose index with melanoma prevalence and all-cause mortality: insights from cross-sectional NHANES 1999–2018 data and an external hospital-based dataset
Source: Front Nutr. 2026 Mar 18;13:1726865. doi: 10.3389/fnut.2026.1726865 (PMC13038597; doi:10.3389/fnut.2026.1726865)
Supplement: Supplementary Table 2 — Sensitivity analysis of multiple imputation (m = 20) for TyG index and melanoma. [file Table_2.docx]

**Supplementary Table 2 Sensitivity analysis of multiple imputation (m=20) for TyG index and melanoma**

|  | Model1 |  | Model2 |  | Model3 |  |
| --- | --- | --- | --- | --- | --- | --- |
| TyG | HR (95%CI) | *P* value | HR (95%CI) | *P* value | HR (95%CI) | *P* value |
| T1 | reference |  |  |  |  |  |
| T2 | 1.707(1.576-1.838) | <0.01 | 1.011(0.892-1.130) | 0.858 | 0.987(0.860-1.114) | 0.841 |
| T3 | 2.601(2.459-2.744) | <0.01 | 1.329(1.201-1.458) | <0.010 | 1.100(0.937-1.264) | 0.252 |

Notes: Sensitivity analysis using weighted cox regression analysis with multiple imputation (m = 20).
