# Supplementary material for: Machine learning-based association analysis of triglyceride-glucose index with melanoma prevalence and all-cause mortality: insights from cross-sectional NHANES 1999–2018 data and an external hospital-based dataset
Source: Front Nutr. 2026 Mar 18;13:1726865. doi: 10.3389/fnut.2026.1726865 (PMC13038597; doi:10.3389/fnut.2026.1726865)
Supplement: Supplementary Table 3 — Baseline characteristics of the external cohort. [file Table_3.docx]

**Supplementary Table 3 Baseline characteristics of the external cohort**

|  |  | Alive | Deceased | *p* |
| --- | --- | --- | --- | --- |
| n | level | 255 | 220 |  |
| Age (years) |  | 51.50 (9.76) | 62.93 (9.02) | <0.001 |
| Gender | Female | 123 ( 48.2) | 99 ( 45.0) | 0.540 |
|  | Male | 132 ( 51.8) | 121 ( 55.0) |  |
| Race | Han | 254 ( 99.6) | 220 (100.0) | 1.000 |
|  | Other Race | 1 (0.4) | 0 (0.0) |  |
| BMI (kg/m²) |  | 23.60 (3.18) | 23.69 (3.17) | 0.773 |
| Glucose (mg/dL) |  | 97.66 (23.41) | 98.51 (23.79) | 0.696 |
| TyG |  | 8.83 (0.67) | 8.80 (0.59) | 0.595 |
| Triglyceride (mg/dL) |  | 1.98 (1.51) | 1.82 (1.24) | 0.207 |
| Total Cholesterol (mg/dL) |  | 5.00 (1.01) | 5.05 (0.97) | 0.612 |
| Potassium (mmol/L) |  | 4.10 (0.32) | 4.07 (0.42) | 0.299 |
| Phosphorus (mmol/L) |  | 1.17 (0.16) | 1.16 (0.18) | 0.419 |
| Albumin (g/dL) |  | 4.33 (0.31) | 4.18 (0.36) | <0.001 |
| Globulin (g/dL) |  | 2.78 (0.39) | 2.90 (0.40) | 0.001 |
| Diabetes | No | 235 ( 92.2) | 202 ( 91.8) | 1.000 |
|  | Yes | 20 ( 7.8) | 18 ( 8.2) |  |
| Hypertension | No | 207 ( 81.2) | 171 ( 77.7) | 0.415 |
|  | Yes | 48 ( 18.8) | 49 ( 22.3) |  |
| Survival_Time (months) |  | 60.73 (37.70) | 39.84 (27.44) | <0.001 |

Note: Continuous variables are expressed as mean (standard deviation), while categorical variables are expressed as frequency (%).
