# Supplementary material for: Machine learning-based association analysis of triglyceride-glucose index with melanoma prevalence and all-cause mortality: insights from cross-sectional NHANES 1999–2018 data and an external hospital-based dataset
Source: Front Nutr. 2026 Mar 18;13:1726865. doi: 10.3389/fnut.2026.1726865 (PMC13038597; doi:10.3389/fnut.2026.1726865)
Supplement: Supplementary Table 4 — Number of deaths (or events) by TyG index stratification levels. [file Table_4.docx]

**Supplementary Table 4 Number of deaths (or events) by TyG index stratification levels**

| TyG_tertile | All | Death | Case fatality rate (%) | *P* value |
| --- | --- | --- | --- | --- |
| T1 | 7138 | 564 | 7.9 | <0.001 |
| T2 | 7129 | 1007 | 14.13 |  |
| T3 | 7134 | 1330 | 18.64 |  |
