# Supplementary material for: Machine learning-based association analysis of triglyceride-glucose index with melanoma prevalence and all-cause mortality: insights from cross-sectional NHANES 1999–2018 data and an external hospital-based dataset
Source: Front Nutr. 2026 Mar 18;13:1726865. doi: 10.3389/fnut.2026.1726865 (PMC13038597; doi:10.3389/fnut.2026.1726865)
Supplement: Supplementary Table 5 — Comparison of calibration indicators (Brier Score) of each model in training and test sets. [file Table_5.docx]

## Supplementary Table 5 Comparison of calibration indicators (Brier Score) of each model in training and test sets

| Dataset | Model Name | Brier Score | Ranking | Calibration Quality |
| --- | --- | --- | --- | --- |
| Training Set | LightGBM | 0.0134 | 1 | Excellent |
|  | XGBoost | 0.0138 | 2 | Excellent |
|  | DT | 0.0375 | 3 | Excellent |
|  | LR | 0.1784 | 4 | Fair |
|  | EN | 0.1793 | 5 | Fair |
|  | Lasso | 0.1795 | 6 | Fair |
|  | RR | 0.1817 | 7 | Fair |
| Test Set | XGBoost | 0.0174 | 1 | Excellent |
|  | LightGBM | 0.0205 | 2 | Excellent |
|  | DT | 0.0685 | 3 | Good |
|  | RR | 0.1980 | 4 | Fair |
|  | EN | 0.1990 | 5 | Fair |
|  | Lasso | 0.1999 | 6 | Fair |
|  | LR | 0.2002 | 7 | Poor |

**Note**: The Brier score measures the mean squared error between the model's predicted probability and the actual event rate, with a value range of 0~1. A smaller value indicates better calibration quality.
