# Supplementary material for: Machine learning-based association analysis of triglyceride-glucose index with melanoma prevalence and all-cause mortality: insights from cross-sectional NHANES 1999–2018 data and an external hospital-based dataset
Source: Front Nutr. 2026 Mar 18;13:1726865. doi: 10.3389/fnut.2026.1726865 (PMC13038597; doi:10.3389/fnut.2026.1726865)
Supplement: Supplementary Table 6 — PPV and NPV of each model at preset threshold (0.5). [file Table_6.docx]

## **Supplementary Table 6** PPV and NPV of each model at preset threshold (0.5)

| Model Type | Models | PPV | NPV |
| --- | --- | --- | --- |
| Tree Model | XGBoost | 0.9964 | 0.9876 |
|  | LightGBM | 0.9946 | 0.9931 |
|  | DT | 0.9305 | 0.9862 |
| Linear Model | LR | 0.7228 | 0.7510 |
|  | RR | 0.7208 | 0.7519 |
|  | EN | 0.7204 | 0.7481 |
|  | Lasso | 0.7181 | 0.7459 |

**Note**: PPV (Positive Predictive Value) refers to the proportion of samples predicted as positive by the model that are actually positive; NPV (Negative Predictive Value) refers to the proportion of samples predicted as negative by the model that are actually negative.
